# Lyme disease transmission by severely impaired ticks

**DOI:** 10.1098/rsob.210244

**Published:** 2022-02-16

**Authors:** Jan Perner, Matej Kucera, Helena Frantova, Veronika Urbanova, Petr Kopacek, Radek Sima

**Affiliations:** Institute of Parasitology, Biology Centre of the Czech Academy of Sciences, Ceske Budejovice 37005, Czech Republic

**Keywords:** Lyme disease, borreliosis, *Borrelia*, tick, transmission, tRNA synthetase

## Abstract

It has been demonstrated that impairing protein synthesis using drugs targeted against tRNA amino acid synthetases presents a promising strategy for the treatment of a wide variety of parasitic diseases, including malaria and toxoplasmosis. This is the first study evaluating tRNA synthetases as potential drug targets in ticks. RNAi knock-down of all tested tRNA synthetases had a strong deleterious phenotype on *Ixodes ricinus* feeding. Our data indicate that tRNA synthetases represent attractive, anti-tick targets warranting the design of selective inhibitors. Further, we tested whether these severely impaired ticks were capable of transmitting *Borrelia afzelii* spirochaetes. Interestingly, biologically handicapped *I. ricinus* nymphs transmitted *B. afzelii* in a manner quantitatively sufficient to develop a systemic infection in mice. These data suggest that initial blood-feeding, despite the incapability of ticks to fully feed and salivate, is sufficient for activating *B. afzelii* from a dormant to an infectious mode, enabling transmission and dissemination in host tissues.

## Graphical abstract

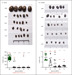

## 1. Background

Lyme borreliosis is the most common vector-borne disease in Europe and the USA. It is caused predominantly by the spirochaete *Borrelia burgdorferi* in the United States or by *Borrelia afzelii, Borrelia garinii* and *B. burgdorferi* in Europe. Currently, there is no human vaccine to prevent borreliosis. The traditional approaches target antigens on the surface of *Borrelia*, such as OspA [1–3], but alternative vaccination strategies targeting tick proteins that *Borrelia* requires for effective transmission to the mammalian host have also been proposed. As ticks are armed with a unique repertoire of salivary molecules displaying anticoagulation, anti-inflammatory and immunomodulatory activities [4–6], it was suggested that tick-transmitted pathogens (TBE, *Borrelia* spp.) exploit the pharmacological activity of tick saliva to colonize and persist in the vector, and support the transmission from the vector to the vertebrate host [7]. Therefore, many researchers performed vaccination studies to block *Borrelia* transmission through the primary neutralization of tick salivary proteins [8–10]. Recently, the first mRNA anti-tick vaccine targeting 19 *Ixodes scapularis* salivary proteins has been tested. This vaccine elicited an antibody response, which consequently induced an immediate inflammatory reaction in the guinea pigs in response to tick attachment. Such a response could help to alert the host shortly after tick attachment, thus facilitating early removal of the tick and preventing transmission of *Borrelia* [11].

It has been documented that *Borrelia* transmission is enhanced in the presence of salivary proteins [7,12,13]. This convention stems from experiments involving co-injection of *Borrelia* with homogenates of tick salivary glands. However, most of these experiments either overestimated the amount of homogenate used, exceeding physiological amounts of saliva produced during *in vivo* feeding [14], or used a homogenate of salivary glands derived from the tick at a different feeding time-point [13]. As the repertoire of salivary gland transcripts and encoded proteins change dramatically during feeding [15,16], time concurrence of salivary gland origin is of importance to mirror the real situation at the feeding site. As a matter of fact, previous exposure to even full feeding of non-infected ticks does not prevent *Borrelia* transmission [17], indicating that antigens present in tick saliva are not protective against *Borrelia* transmission.

During the past decade, it has been demonstrated that impairing protein synthesis using drugs targeted against tRNA amino acid synthetases presents a promising strategy for treating a wide variety of parasitic diseases (see [18] for review). Thus, a number of drug candidates were tested against protozoan parasites causing malaria [19], toxoplasmosis [20], cryptosporidiosis [21] as well as multicellular helminth parasites causing filariasis or schistosomiasis [22].

In this work, we selected four tRNA synthetases—two from each aminoacyl tRNA synthetase class [23], valyl- (val-) and leucyl- (leu-) tRNA synthetases of class I and asparaginyl- (asn-) and lysyl- (lys-) tRNA synthetases of class II—and evaluated their essentiality in *Ixodes ricinus* ticks using RNAi-mediated silencing. RNAi knock-down of all tested tRNA synthetases had a strong deleterious phenotype on tick feeding capacity. A fascinating question we addressed in this study was whether or not these severely impaired ticks were still capable of transmitting *B. afzelii* spirochaetes to naive mice.

## 2. Material and methods

### 2.1. Laboratory animals

*Ixodes ricinus* larvae, nymphs and adults were obtained from the breeding facility of the Institute of Parasitology, Biology Centre, Czech Academy of Sciences. To prepare infected *I. ricinus* nymphs, the larvae were fed on *B. afzelii* CB43 infected mice and allowed to moult to nymphs. Nymphs were considered to be infected if greater than 90% of them were PCR positive (for details, see [24]). Inbred, pathogen-free C3H/HeN mice (Jackson Laboratory, Germany) were used for the pathogen transmission experiment.

### 2.2. Nucleic acid isolation and cDNA preparation

Total DNA was isolated from murine tissues using a NucleoSpin Tissue Kit (Macherey-Nagel, Germany) according to the manufacturer's protocol.

Total RNA was extracted from nymphs and adult females using a NucleoSpin RNA Kit (Macherey-Nagel) according to the manufacturer's protocol. Isolated RNA was reverse transcribed into cDNA using the Transcriptor High Fidelity cDNA Synthesis Kit (Roche, Germany). All cDNA preparations were prepared in biological triplicates.

### 2.3. RNAi

Four tRNA synthetases were identified as RNAi targets in our previously described *I. ricinus* midgut transcriptome [25], namely Asparaginyl-tRNA synthetase (*Ir*Asn-RS; GenBank JAP73321), Valyl-tRNA synthetase (*Ir*Val-RS; GenBank MBK3721917), Leucyl-tRNA synthetase (*Ir*Leu-RS; GenBank JAP71855) and Lysyl-tRNA synthetase (*Ir*Lys-RS; GenBank JAP72320). A 328 bp fragment of *Ir*Asn-RS, a 560 bp fragment of *Ir*Val-RS, a 420 bp fragment of *Ir*Leu-RS and a 380 bp fragment of *Ir*Lys-RS was each amplified from *I. ricinus* midgut-specific cDNA using primers listed in electronic supplementary material, table S1, and cloned into plasmid pll10. Respective dsRNA fragments were synthesized as described previously [26]. Each dsRNA preparation (1.5 µg μl^–1^) was injected (345 nl or 32.2 nl) into the haemocoel of *I. ricinus* adult females or nymphs, respectively, using Nanoinject II (Drummond). After 3 days of rest in a humid chamber at room temperature, ticks were fed on rabbits or C3H/HeN mice. The level of gene silencing was measured by RT-qPCR.

### 2.4. Transmission and quantification of *Borrelia* in mouse organs

*Borrelia afzelii*-infected nymphs injected with dsRNA were fed on naive C3H/HeN mice (three nymphs per mouse) for 3 days and then forcibly removed. Each experiment was carried out with five mice per dsRNA group. Engorgement weights and feeding successes were recorded for each individual nymph.

Four weeks after the challenge by infected tick nymphs, the total spirochaete load in murine tissues was determined by quantitative real-time PCR (qPCR) as described previously [24]. The spirochaete burden in murine tissues was expressed as the number of spirochaetes per 10^6^ murine ß-actin copies.

cDNAs from silenced nymphs and adult females served as templates for the quantitative expression analyses by relative qPCR. Relative expressions of *Ir*Asn-RS, *Ir*Val-RS, *Ir*Leu-RS and *Ir*Lys-RS were normalized to *I. ricinus* elongation factor 1 using the ΔΔCt method [27].

### 2.5. Quantitative protein determination by Bradford assay

*Ixodes ricinus* nymphs were fed on mice for 48 h. Forcibly detached nymphs were opened by micro-dissection, and a pair of salivary glands was removed, washed in ice-cold PBS and placed into ice-cold PBS containing a tablet of protease inhibitor mix (Thermo Scientific A32963). Seven pairs of salivary glands were homogenized in 50 µl of the buffer using a 29G insulin needle and three freeze/thaw cycles. The homogenate was clarified by centrifugation at 15 000*g*, 20 min, 4°C. The quantity of soluble protein present in the extract was determined in a Bradford assay by absorbance at 595 nm (A_595_), with a dilution of the sample to a value of A_595_ ∼ 0.2.

### 2.6. Preparation of salivary glands for microscopy

Silenced *I. ricinus* nymphs were fed on mice for 72 h. A pair of salivary glands from 10 nymphs was dissected, mounted in DABCO mounting medium and immediately inspected under the Olympus BX53 microscope (darkfield, magnification 10×). The area of 10 representative acini from individual salivary glands was measured using the ImageJ program. The size of the acini in silenced nymphs was compared with the GFP control group.

### 2.7. Statistical analysis

Data were analysed by GraphPad Prism 6 for Windows, v. 6.04, and an unpaired Student's *t*-test was used for evaluation of statistical significance. A *p*-value of less than 0.05 was considered statistically significant. Error bars in the graphs show the standard errors of the means.

## 3. Results

### 3.1. tRNA-synthetases are excellent RNAi targets for effective impairment of tick feeding

**Figure 1.**
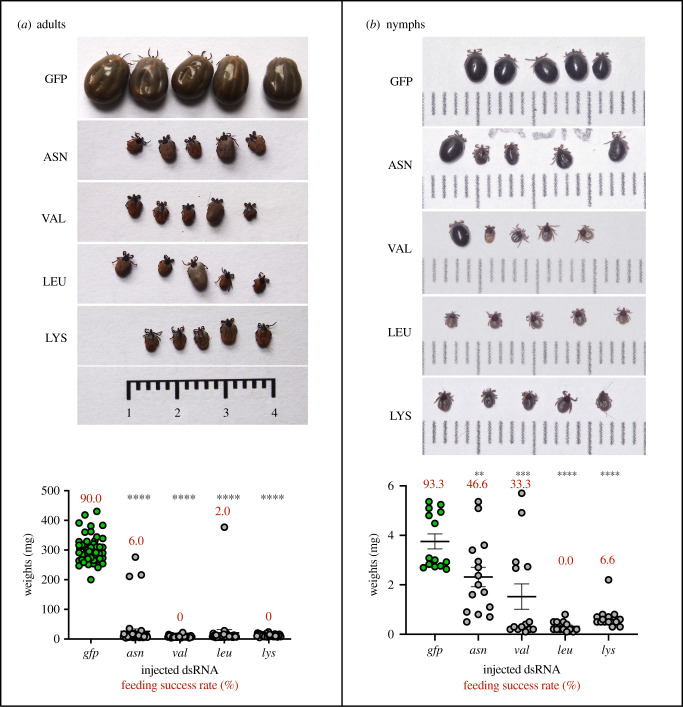
RNAi effect on tick feeding capacity. Silencing of the Asparaginyl-tRNA synthetase, Valyl-tRNA synthetase, Leucyl-tRNA synthetase and Lysyl-tRNA synthetase and their impact on engorgement weight in *I. ricinus* (*a*) adult females and (*b*) nymphs. Photographic images of five individual representative ticks after 8 days (adults) and 3 days (nymphs) of feeding are shown on top. Bottom graphs show weights of all ticks after 8 days (adults) and 3 days (nymphs) of feeding. Bars indicate standard errors of means. Success rate indicates the percentage of how many ticks managed to fully engorge.

Tick feeding is mediated by immense metabolic transformation from a ‘dormant’ state into a highly processive state [28]. To enable tick attachment yet diminish the expression of tick proteinaceous products to physiologically ‘freeze’ the ticks, we selected four genetic knock-downs with a severe impact on the feeding of adult *I. ricinus* females. Mutant ticks were produced by RNAi-mediated silencing of four transcripts encoding enzymes involved in proteosynthesis, namely *Ir*Asn-RS, *Ir*Val-RS, *Ir*Leu-RS and *Ir*Lys-RS. Only 0–6% of ticks injected with respective dsRNAs were able to finish feeding, and their average weights were 25.5, 8.4, 20.8 and 11.8 mg, respectively. In comparison, 90% of *gfp*-dsRNA injected females completed feeding, and their average weight was 303.2 mg (figure 1*a*). These transcripts were clearly good RNAi targets, producing biologically handicapped ticks with severe feeding disabilities. While these ticks remained attached to the host for days and were viable, they did not display any feeding progression typical for ticks (slow and rapid feeding phases). Therefore, we denoted these ticks as ‘zombie ticks’.

### 3.2. RNAi of tRNA-synthetases severely impairs feeding of tick nymphs and retards the development of their salivary glands

**Figure 2.**
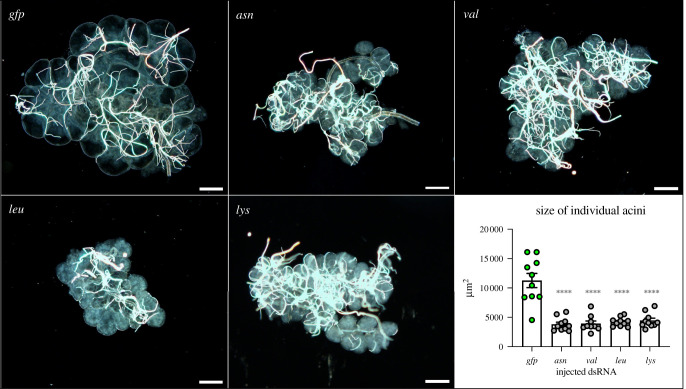
Effect of tRNA synthetase silencing on salivary gland morphology. Microscopic images of representative salivary glands dissected from silenced *I. ricinus* nymphs fed for 3 days. Scale bars represent 100 µm. The graph represents the size of 10 individual acini in each experimental group. The area of acini was measured using the ImageJ program. Bars indicate standard errors of means. ****, *p* < 0,0001.

To confirm whether *I. ricinus* nymphs are sensitive to RNAi silencing of tRNA synthetases, we injected dsRNAs into unfed nymphs and monitored their capacity to feed on a host. Indeed, nymphs, a key developmental stage of *I. ricinus* ticks that transmit Lyme borreliosis, were severely impaired by silencing tRNA-synthetases transcripts (figure 1*b*). While RNAi of *asn*- and *val-*tRNA synthetase reduced the capacity of ticks to engorge only partially (46.6% and 33.3%, respectively), no nymphs engorged in *leu*-, and only 6.6% of *lys*-tRNA synthetase silenced nymphs managed to engorge. On average, RNAi silenced nymphs had 2.3, 1.5, 0.3 and 0.7 mg, compared to 3.8 mg of *gfp* control (figure 1*b*). These ticks thus clearly imbibed a reduced amount of blood. To inspect whether RNAi of tRNA synthetases has any impact on tick salivation, we analysed salivary gland morphology of the RNAi-silenced nymphs and quantified soluble protein content within. Indeed, disrupted proteosynthesis mediated by RNAi of tRNA synthetases resulted in reduced saliva production. A pair of salivary glands from silenced *I. ricinus* nymphs fed for 48 h contained approximately 3–12 fold lower concentrations of soluble proteins than control ticks (table 1). Moreover, salivary glands from silenced nymphs showed aberrant morphology. In comparison with controls, the size of individual acini was significantly reduced, and the acinar content was markedly dense, suggesting substantial changes in salivary gland physiology (figure 2).

**Table 1.** The concentration of soluble proteins in salivary glands of tRNA synthetase silenced nymphs.

injected dsRNA	ng of protein per SG pair
*gfp*	920.8 ± 306.1, *n* = 3
*asn*	89.19 ± 48.77, *n* = 3
*val*	74.90 ± 69.72, *n* = 3
*leu*	359.2 ± 25.88, *n* = 3
*lys*	310.0 ± 68.45, *n* = 3

### 3.3. Assessment of *Borrelia* transmission by ‘zombie’ tick nymphs

**Figure 3.**
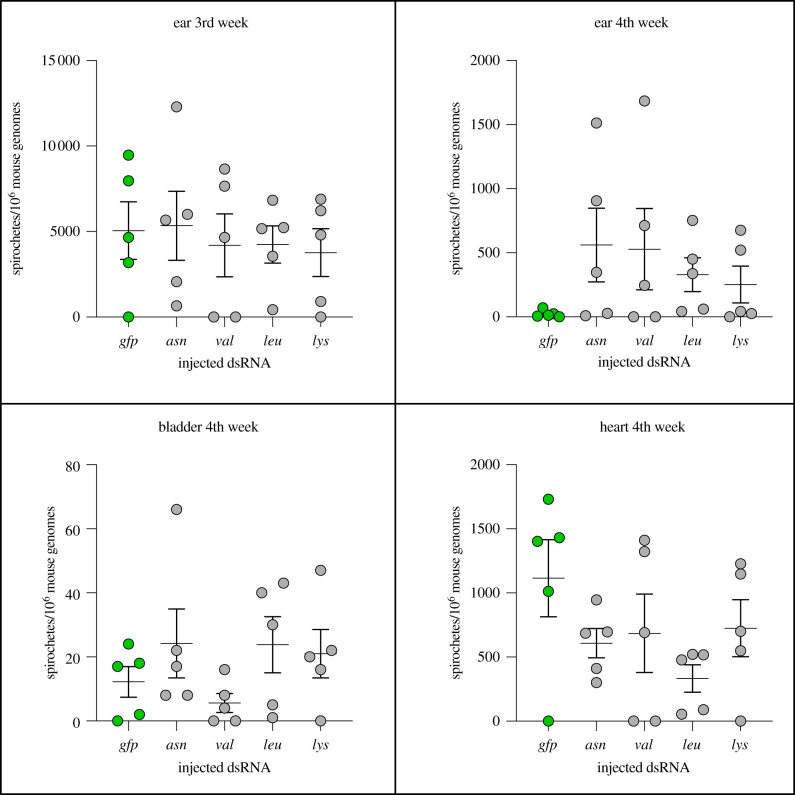
Quantitative evaluation of *Borrelia* spirochaete loads in murine tissues after transmission by ‘zombie’ ticks. Each data point represents the number of *B. afzelii* spirochaetes per 10^6^ murine genomes in the individually analysed ear, bladder and heart biopsy specimens. Bars indicate standard errors of means. Differences in spirochaete load between mice infested with *asn*-, *val*-, *leu*-, *lys*- and *gfp*-dsRNA were not statistically significant.

The silenced *I. ricinus* nymphs managed to attach, but except for *asn*-tRNA synthetase KD, ticks gained very little or no weight during 3 days of feeding, indicating severely compromised blood uptake. The RNAi silencing of tRNA synthetase transcripts was evoked in unfed ticks, but the high efficiency of KD was also confirmed upon nymphal feeding (electronic supplementary material, S1 figure). Whether these ‘zombie’ tick nymphs would be capable of transmitting *Borrelia* spirochaetes was further addressed. By feeding tick larvae on *B. afzelii*-infected mice, infectious nymphs were produced with a greater than 90% infection rate [24]. These nymphs were micro-injected with respective dsRNAs and were allowed to feed on naive mice for 3 days. After 3 days, ‘zombie’ ticks were removed, and mice were left to develop the infection for four weeks. All mice, irrespective of injected dsRNA (*asn*-dsRNA, *val-*dsRNA, *leu*-dsRNA, and *lys*-dsRNA or *gfp-*dsRNA), were populated by quantitatively identical amount of *B. afzelii* spirochaetes in the mouse ear, bladder and heart (figure 3). These data indicate that *B. afzelii* spirochaetes are able to migrate from *I. ricinus* nymphs to a vertebrate host with minimal tick physiological contribution.

## 4. Discussion

Ticks possess very complex feeding biology similar to other hematophagous arthropods. However, they have distinct features. Their ability to imbibe a vast amount of blood over a long period of time explains their remarkable success as disease vectors. *Ixodes ricinus* is the primary vector of tick-borne encephalitis (TBE) virus and Lyme borreliosis spirochaetes in Europe. While the TBE can be successfully controlled by vaccination, a human vaccine against borreliosis is not currently available. In 1998, the FDA approved a new recombinant Lyme vaccine, LYMErix, targeting the outer surface protein A (OspA). Nevertheless, low demand for the vaccine, combined with mounting lawsuits from patients, led to the manufacturer pulling out of the market in 2002. Although the second generation of OspA-based vaccines is currently being tested [2,3,29], alternative strategies for preventing Lyme borreliosis have been suggested. Tick molecules that the *Borrelia* spirochaetes require for mammalian host infection have been proposed as promising targets [30,31]. Ticks secrete a wide range of biologically active proteins into the feeding site. It is assumed that most of these molecules play essential roles in modulating host defence mechanisms, and are critical for effective tick attachment and engorgement [6]. Many investigators work with the hypothesis that the immunomodulatory effect of tick saliva is also exploited by tick-transmitted pathogens, including *Borrelia*, during their transmission. First studies demonstrated that injection of *Borrelia* spirochaetes into mice, together with *I. ricinus* or *I. scapularis* salivary gland extract (SGE), increased the level of spirochaete burden in mice and enhanced the infection of feeding ticks [13,32]. The presence of SGE has also been reported to suppress the production of pro-inflammatory cytokines, reducing the production of anti-microbial molecules involved in phagocytosis (superoxide, nitric oxide) and inhibiting the killing of *B. afzelii* by macrophages *in vitro* [33]. Despite the indisputable value of these pioneering *in vitro* data, new reassessments regarding a systemic *in vivo* framework might reveal further details. Most of the early studies employed SGE, or pilocarpine-induced saliva from half-fed adult females. It has been reported that the pilocarpine concentration in tick saliva is much higher than the minimum required for the activity of this agent. Due to the known effects of pilocarpine on smooth muscle and immune cells, caution should be taken when interpreting results obtained with this type of preparation [34].

While the pioneering works on salivary-assisted transmission used 10–50% of species-specific SGE, representing 50–250 ng of protein [35], later SAT studies used dozens of micrograms of SGEs [13,32], despite the fact that a pair of salivary glands from an *I. ricinus* nymph fed for 48 h contains about one microgram of soluble protein (table 1). Therefore, the protein concentrations used in experimental infections vastly exceeded physiological amounts of proteins naturally delivered into the host by the tick-bite. The experimental set-up thus only remotely resembled the natural environment for *Borrelia* transmission. Later studies were focused on the identification of specific tick molecules involved in pathogen transmission. Various salivary molecules that modulate T-cell activation (Salp15), complement (Isac, Salp20, TSLPI), release of histamine (tHRF) and other components of the host immune system have been described [8–10,36]. RNAi-silencing of these single salivary molecules was reported to impair *Borrelia* transmission.

Recently, we have challenged the salivary route of transmission for the *B. afzelii* and European Lyme disease vector *I. ricinus* [24] and provided new data which supported the original concept of direct ‘gut-to-mouth’ route of the host infection by *Borrelia* spirochaetes suggested by Willy Burgdorfer and others [37,38]. Our study challenged the role of *I. ricinus* saliva in the infectivity and survival of *B. afzelii* spirochaetes. We suggested that the main requirement for *B. afzelii* survival and successful host colonization is a complex remodelling of surface antigens and not the immunomodulatory effect of tick saliva. *B. afzelii* was depicted as an independent, motile bacterium that actively migrated from the tick midgut towards the host tissues [24].

The current study provides an additional piece of evidence that *I. ricinus* salivary glands and saliva proteins do not play a decisive role in *B. afzelii* transmission, suggesting that transmission of spirochaetes happens independently of continuous tick feeding (blood uptake and salivation). RNAi-mediated knock-down of four essential tRNA synthetases resulted in severe phenotypes regarding the progression of *I. ricinus* feeding. However, *B. afzelii* spirochaetes were transmitted in comparable quantities to the normally feeding dsRNA-mock control. We argue that, during tick feeding, *I. ricinus* ticks serve as a mere vehicle that needs to (i) mediate the connection with host tissues using hypostome mechanics and early saliva, and (ii) take up initial blood meal to trigger the infectivity of *B. afzelii* spirochaetes. This all facilitates an essential bridging of tick midgut with vertebrate bloodstream, enabling *Borrelia* to transverse between these two hosts. We suggest that spirochaetes then actively transverse into the host independently of concomitant processes inherent to tick feeding. We argue that the success of *B. afzelii* dissemination in the host is mainly determined by their ‘infectivity mode’, a shift undertaken while in the tick midgut, rather than continuous support of tick salivation.

We suggest that similar studies involving other tick and *Borrelia* species should be conducted to answer the question of whether our data are specific to the European case of *B. afzelii* and *I. ricinus*, or a general phenomenon that tick saliva is dispensable for successful *Borrelia* transmission.

## 5. Conclusion

In this study, we have:

(1) Identified and validated four tick tRNA synthetases across two developmental stages of *I. ricinus*. Using RNA interference, we have demonstrated that tRNA synthetases represent promising anti-tick targets, which warrant the design of selective inhibitors.
(2) Demonstrated that silencing of tRNA synthetases severely impairs *I. ricinus* feeding and retards the normal function of tick tissues, including salivary glands, which is best exemplified by *leu*-tRNA KD ticks. The *leu*-tRNA KD nymphs gained virtually no weight from blood-feeding (0.3 mg compared to 0.24 mg of unfed nymphs). Interestingly, these severely handicapped nymphs did not lose their capacity to transmit *B. afzelii* spirochaetes. These data indicate that tick physiology, including salivary gland products, is dispensable for the dissemination of *B. afzelii* spirochaetes.
(3) Expressed our opinion that arrest in tick feeding is not a sufficient phenotype to prevent *B. afzelii* transmission. This, to a large extent, negates the ambition to prevent Lyme borreliosis by antibody-mediated targeting of *I. ricinus* salivary proteins.

## Data accessibility

This article has no additional data.
